# Comparative Pharmacological and Pharmaceutical Perspectives on Antidiabetic Therapies in Humans, Dogs, and Cats

**DOI:** 10.3390/pharmaceutics17091098

**Published:** 2025-08-23

**Authors:** Iljin Kim, Jang-Hyuk Yun

**Affiliations:** 1 Department of Pharmacology and Program in Biomedical Science and Engineering, Inha University College of Medicine, Incheon 22212, Republic of Korea; ijk@inha.ac.kr; 2 Research Center for Controlling Intercellular Communication, Inha University College of Medicine, Incheon 22212, Republic of Korea; 3 College of Veterinary Medicine and Institute of Veterinary Science, Kangwon National University, Chuncheon 24341, Republic of Korea

**Keywords:** diabetes mellitus, endocrine disorder, species-specific antidiabetic therapies, canine, feline, one health approach

## Abstract

**Background/Objectives:** Diabetes mellitus (DM) is an increasingly prevalent endocrine disorder affecting humans and companion animals. Type 1 DM (T1DM) and type 2 DM (T2DM) are well characterized in humans, and canine DM most often resembles T1DM, marked by insulin dependence and β-cell destruction. Conversely, feline DM shares key features with human T2DM, including insulin resistance, obesity-related inflammation, and islet amyloidosis. This review provides a comprehensive comparative analysis of antidiabetic therapies in humans, dogs, and cats, focusing on three core areas: disease pathophysiology, pharmacological and delivery strategies, and translational implications. In human medicine, a wide array of insulin analogs, oral hypoglycemic agents, and incretin-based therapies, including glucagon-like peptide-1 receptor agonists (liraglutide) and sodium-glucose cotransporter-2 inhibitors (empagliflozin), are available. Veterinary treatments remain limited to species-adapted insulin formulations and off-label use of human drugs. Interspecies differences in gastrointestinal physiology, drug metabolism, and behavioral compliance influence therapeutic efficacy and pharmacokinetics. Recent innovations, such as microneedle patches for insulin delivery and continuous glucose monitoring systems, show promise in humans and animals. Companion animals with naturally occurring diabetes serve as valuable models for preclinical testing of novel delivery platforms and long-acting formulations under real-world settings. While these technologies show potential, challenges remain in regulatory approval and behavioral adaptation in animals. **Conclusions:** Future research should prioritize pharmacokinetic bridging studies, veterinary-specific formulation trials, and device validation in animal models. By highlighting shared and species-specific characteristics of DM pathogenesis and treatment, this review advocates a One Health approach toward optimized antidiabetic therapies that benefit human and veterinary medicine.

## 1. Introduction

Diabetes mellitus (DM) is a chronic metabolic disorder characterized by persistent hyperglycemia resulting from defects in insulin secretion, insulin action, or both [1,2,3]. Over the past few decades, the global prevalence of diabetes reached epidemic proportions, posing a major public health and economic burden worldwide [4,5]. According to the 12th edition of the International Diabetes Federation Diabetes Atlas (2023), approximately 537 million adults (20–79 years) were living with diabetes globally in 2021. This number is expected to rise to 643 million by 2030 and 783 million by 2045 [6]. Diabetes significantly increases the risk of severe complications, such as cardiovascular disease, kidney failure, neuropathy, retinopathy, and premature mortality, underscoring the urgency of optimizing the therapeutic strategies [7,8,9].

Importantly, diabetes is not a condition unique to humans. It is increasingly diagnosed in companion animals, particularly dogs and cats, mirroring trends observed in human populations. According to the Banfield Pet Hospital State of Pet Health Report (2016), as cited in the review by Moshref et al., the incidence of canine DM increased by approximately 80% between 2006 and 2015 in the United States [10]. This growing prevalence is closely linked to shared risk factors such as aging, obesity, sedentary lifestyles, and even dietary patterns in domestic settings. Canine diabetes most commonly resembles human type 1 DM (T1DM), characterized by insulin dependence and β-cell destruction, whereas feline diabetes typically presents as a type 2-like condition involving insulin resistance and β-cell dysfunction [11,12].

As veterinary care improves and companion animals live longer, the clinical burden of DM in these species continues to rise. However, veterinary therapeutic options remain severely limited. Currently, no oral hypoglycemic agents have been approved for use in dogs or cats [13]. Glycemic monitoring also presents challenges, as continuous glucose monitoring (CGM) systems are rarely adapted for veterinary use [14]. Additionally, the off-label use of human-approved medications often results in suboptimal efficacy or adverse effects due to interspecies differences in pharmacokinetics and treatment compliance behaviors [15,16].

Given the physiological, pathological, and environmental parallels between humans and companion animals, a comparative evaluation of antidiabetic therapies across species is both timely and necessary. Dogs and cats with naturally occurring DM provide valuable translational models that aid veterinary drug development and human diabetes research, particularly in areas such as insulin pharmacokinetics, formulation design, and behavioral adherence to therapy [11].

In human medicine, a broad spectrum of antidiabetic therapies has been developed, including rapid- and long-acting insulin analogs, oral hypoglycemic agents (e.g., metformin, sodium-glucose cotransporter-2 [SGLT2] inhibitors), and incretin-based therapies (e.g., glucagon-like peptide-1 [GLP-1] receptor agonists) [17,18,19]. Contrastingly, veterinary treatments often rely on a limited number of insulin formulations (including porcine lente insulin [Vetsulin]) and the off-label use of human medications, which lack species-specific pharmacodynamic validation [20,21].

Recent studies explored novel therapeutic approaches with translational potential. For instance, glucose-responsive photodynamic analgesic gels demonstrated localized efficacy in diabetic wound healing [22]. Observational studies reported associations between low vitamin D levels and increased cardiovascular risk in patients with type 2 DM (T2DM), although trials on dietary supplements yielded inconsistent results [23,24,25]. However, most of these innovations remain underexplored in veterinary medicine.

Despite growing interest, a significant gap persists in the understanding of how the mechanisms, absorption, metabolism, formulation challenges, and delivery strategies of antidiabetic drugs differ across species. Previous studies largely examined these issues in isolation—focusing on either human or veterinary contexts—without a systematic, cross-species comparison within the unified One Health framework [16,26]. Furthermore, species-specific barriers, including differences in gastrointestinal (GI) physiology, hepatic enzyme activity, palatability, and dosing behavior, present critical obstacles to effective therapy but are often overlooked in existing reviews.

To our knowledge, this is the first comprehensive review to integrate pharmacological mechanisms, pharmacokinetic profiles, and formulation strategies of antidiabetic therapies across humans, dogs, and cats. In addition to reviewing established drug classes, this study incorporates emerging delivery technologies—microneedle patches, buccal films, and CGM systems—relevant to both human and veterinary medicine [14,27,28,29,30]. Moreover, the review uniquely emphasizes the impact of regulatory gaps, species-specific compliance challenges, and real-world translational applicability, which are rarely addressed in previous studies.

By identifying these cross-species gaps and synthesizing current knowledge, this review provides an integrated perspective to support the development of optimized, clinically effective, and translationally relevant antidiabetic therapies within a One Health paradigm.

## 2. Pathogenesis of DM in Humans, Dogs, and Cats

### 2.1. Type 1 vs. Type 2 Distribution Across Species

#### 2.1.1. Brief Description

DM comprises a group of metabolic disorders characterized by chronic hyperglycemia resulting from impaired insulin secretion, insulin action, or both [31]. The two major clinical forms—T1DM and T2DM—differ significantly in pathogenesis and distribution across species.

#### 2.1.2. Key Findings

In humans, both T1DM (autoimmune-mediated β-cell destruction leading to absolute insulin deficiency) and T2DM (insulin resistance with relative insulin deficiency) are prevalent. T1DM typically manifests in childhood or adolescence, while type 2 is more common in adults and is associated with obesity, sedentary lifestyle, and aging [32,33]. In dogs, most DM cases resemble T1DM, with patients exhibiting insulin dependence due to significant β-cell loss. However, the underlying pathogenesis is less frequently autoimmune and more often idiopathic or secondary to pancreatitis [11,34]. Canine diabetes typically develops in middle-aged to older dogs and is irreversible, necessitating lifelong insulin therapy [35]. Conversely, diabetes in cats more closely resembles human T2DM, characterized by insulin resistance and progressive β-cell dysfunction [36]. Obesity, amyloid deposition in pancreatic islets, and chronic inflammation contribute to disease onset and progression [3,37]. Notably, feline diabetes may be transient or even reversible with early and aggressive glycemic control [38].

#### 2.1.3. Academic Discussion

The pathophysiological characteristics of DM differ markedly across humans, dogs, and cats, particularly in terms of insulin dependence, β-cell pathology, and potential for remission [1]. These interspecies differences are shaped by distinct islet architectures, immune responses, and environmental exposures. For example, autoimmune β-cell destruction is the hallmark of human T1DM, whereas in dogs, β-cell loss is more commonly associated with idiopathic etiology or pancreatitis-related mechanisms rather than autoimmunity [34,35]. Contrastingly, islet amyloidosis—a feature prominently seen in humans with T2DM—is also observed in feline diabetes, but is notably absent in dogs, further emphasizing species-specific pathological divergence [11,39].

Feline diabetes is uniquely characterized by obesity-induced insulin resistance and amyloid-associated β-cell dysfunction, closely resembling human T2DM [11]. Importantly, early glycemic control in cats can lead to remission, a phenomenon rarely observed in dogs [40,41]. This underscores the potential reversibility of β-cell stress in certain species and supports the concept of a therapeutic window for β-cell recovery in early-stage diabetes. These insights not only aid veterinary management, but also offer translational relevance for understanding β-cell plasticity in human T2DM.

In humans, gestational DM and assisted reproductive technologies have been linked to long-term metabolic disturbances in offspring, highlighting how prenatal metabolic stressors may contribute to the transgenerational risk of diabetes [42,43,44]. Although such mechanisms are less studied in companion animals, emerging data on reproductive endocrinology in dogs and cats could provide additional comparative insight.

**Figure 1:**
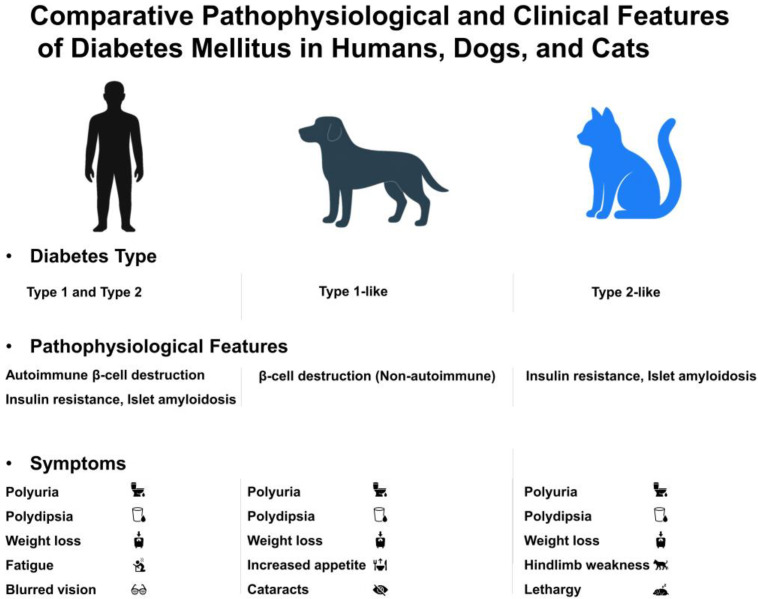
Comparative pathophysiology and clinical features of diabetes mellitus in humans, dogs, and cats. Humans typically present with both type 1 and type 2 diabetes mellitus, whereas dogs primarily exhibit a type 1-like, insulin-dependent form, and cats develop a type 2-like form characterized by insulin resistance and islet amyloidosis. Shared clinical signs include polyuria, polydipsia, and weight loss. Species-specific symptoms, such as fatigue and blurred vision (humans), cataracts and increased appetite (dogs), or hindlimb weakness and lethargy (cats), are also highlighted.

A detailed summary of species-specific diabetic features is presented in Table 1, including dominant diabetes types, pathophysiological hallmarks, hormonal profiles, and clinical symptoms. Furthermore, interspecies differences in pathogenesis, remission potential, and symptomatology are illustrated in Figure 1, which compares diabetes-related traits among humans, dogs, and cats.

### 2.2. Multifactorial Pathogenesis of T2DM

#### 2.2.1. Brief Description

T2DM is now recognized as a multifactorial disease involving numerous interrelated pathogenic mechanisms, many of which are closely linked to targeted treatment strategies [45]. It is the most prevalent form of diabetes in humans and is closely associated with obesity, sedentary lifestyle, and genetic factors.

#### 2.2.2. Key Findings

In humans, the earliest metabolic disturbances typically involve insulin resistance in skeletal muscle, liver, and adipose tissue. This is followed by compensatory hyperinsulinemia by pancreatic β-cells, which temporarily maintains glycemic control. However, progressive β-cell dysfunction—driven by glucotoxicity, lipotoxicity, oxidative stress, and chronic inflammation—eventually leads to inadequate insulin secretion and overt hyperglycemia [46].

In addition to insulin resistance and β-cell failure, other critical pathogenic pathways have been identified. Impaired incretin signaling, particularly reduced GLP-1 and gastric inhibitory polypeptide activity, diminishes glucose-stimulated insulin secretion and contributes to hyperglycemia [47]. Hyperglucagonemia, characterized by inappropriate glucagon secretion, increases hepatic glucose production in both fasting and postprandial states [48]. Adipokine dysregulation, such as low adiponectin and high leptin levels, exacerbates insulin resistance and systemic inflammation [49]. Mitochondrial dysfunction in liver and muscle cells impairs oxidative metabolism and insulin signaling [50]. Gut microbiota dysbiosis alters intestinal barrier function, promotes endotoxemia, and influences systemic metabolic responses [51]. Amyloid deposition in pancreatic islets, largely derived from islet amyloid polypeptide (IAPP), leads to β-cell cytotoxicity and disease progression [52]. Enhanced renal glucose reabsorption due to increased SGLT2 expression sustains hyperglycemia [53]. Finally, hypothalamic insulin resistance can impair central regulation of appetite and energy balance, contributing to metabolic dysregulation [54].

#### 2.2.3. Academic Discussion

The pathogenesis of T2DM is a complex interplay of metabolic, hormonal, and inflammatory pathways that not only progress over time, but also interact to accelerate β-cell dysfunction and systemic insulin resistance. These mechanisms form the basis for current therapeutic targets and help explain the variable treatment responses observed among patients.

Importantly, the manifestation of these pathways shows distinct interspecies variation. In dogs, classic T2DM with obesity-induced insulin resistance is uncommon. However, insulin resistance may occur in specific populations, such as obese intact females or dogs with concurrent endocrine disorders such as hyperadrenocorticism, suggesting a role for hormonal and metabolic crosstalk in canine diabetes development [55,56]. Nonetheless, most dogs with diabetes remain insulin-dependent, aligning more closely with human T1DM.

Conversely, feline diabetes strongly mirrors the human T2DM phenotype in both etiology and progression. Cats exhibit obesity-associated insulin resistance, islet amyloidosis due to IAPP accumulation, and chronic low-grade inflammation, all of which contribute to progressive β-cell dysfunction [3,28]. The potential for diabetic remission in cats—especially with early insulin therapy and dietary management—underscores the importance of β-cell preservation strategies and parallels the reversibility observed in some early-stage human T2DM cases.

Recent preclinical evidence further supports the use of traditional herbal medicines, such as the Liuwei Dihuang decoction, for alleviating β-cell stress and improving insulin sensitivity. These formulations appear to modulate key metabolic pathways, such as PI3K/Akt, which are conserved across species and represent promising translational targets for both human and veterinary medicine [57,58]. Overall, the diverse but overlapping pathophysiological mechanisms of T2DM across species emphasize the need for individualized treatment approaches while also providing opportunities for comparative and translational research. These insights offer a foundation for developing species-specific therapies and for testing novel interventions in veterinary models with high relevance to human disease.

### 2.3. Hormonal Modulators of Glucose Homeostasis

#### 2.3.1. Brief Description

Hormonal regulators such as GLP-1, amylin, and glucagon play central roles in glucose homeostasis and are differentially involved in the pathogenesis of DM across species.

#### 2.3.2. Key Findings

GLP-1 is an incretin hormone secreted by intestinal L-cells. It enhances glucose-stimulated insulin secretion, inhibits glucagon release, delays gastric emptying, and promotes satiety [59]. In human T2DM, GLP-1 secretion or signaling is often impaired [60]. In cats, GLP-1 signaling abnormalities have also been reported, especially in the case of obesity [61]. In dogs, limited data exist; however, GLP-1–based therapies are being explored in experimental settings [62].

Amylin, or IAPP, is co-secreted with insulin by pancreatic β-cells. It suppresses postprandial glucagon secretion and slows gastric emptying. In both humans and cats with T2DM, IAPP aggregates to form amyloid fibrils, which are cytotoxic to β-cells and exacerbate disease progression [3,52]. Such amyloid deposition is not typically observed in dogs [63].

Glucagon, secreted by pancreatic α-cells, promotes hepatic gluconeogenesis and glycogenolysis. In diabetes, inappropriate hyperglucagonemia contributes to fasting and postprandial hyperglycemia [64]. This mechanism is well documented in humans and is presumed to operate similarly in dogs and cats, although direct species-specific data are limited.

#### 2.3.3. Academic Discussion

While GLP-1, amylin, and glucagon pathways are conserved across species, key interspecies differences offer insights for therapeutic translation. For example, islet amyloid formation is prominent in humans and cats, but not in dogs, suggesting species-specific differences in IAPP processing and β-cell vulnerability [63]. This makes feline and human models particularly valuable for studying amyloid-mediated β-cell failure.

GLP-1 signaling defects, common in human and feline T2DM, reduce insulinotropic responses and promote hyperglycemia [59,65]. The lack of robust canine data limits current applications, but the success of GLP-1–based therapies in humans warrants further investigation in veterinary settings. Similarly, the role of hyperglucagonemia in human diabetes may also extend to companion animals, positioning glucagon suppression (via GLP-1 analogs or amylin mimetics) as a potential cross-species therapeutic strategy.

### 2.4. Inflammatory Mediators and Immunometabolic Dysregulation

#### 2.4.1. Brief Description

Chronic low-grade inflammation is a hallmark of T2DM and contributes to insulin resistance and β-cell dysfunction. Inflammatory cytokines and immunometabolic disturbances are increasingly recognized as key modulators in the development and progression of DM across species.

#### 2.4.2. Key Findings

Low-grade systemic inflammation, marked by elevated levels of TNF-α, IL-6, and other pro-inflammatory cytokines, plays a crucial role in human T2DM pathogenesis [66]. These cytokines disrupt insulin signaling and promote β-cell apoptosis. In cats and dogs, obesity-induced inflammation has been linked to insulin resistance and diabetes development [67,68], although species-specific mechanistic details remain less defined.

#### 2.4.3. Academic Discussion

Obesity-associated inflammation appears to be a conserved feature in human, feline, and canine diabetes, but the nature and impact of specific mediators may differ by species. For example, TNF-α and IL-6 interfere with insulin receptor signaling, contributing to both hepatic and peripheral insulin resistance [66]. The intensity, cellular source, and downstream metabolic effects of these cytokines likely vary depending on species, age, and adipose tissue distribution.

Comparative studies investigating the immunometabolic interface in animals with diabetes could uncover novel biomarkers and inform anti-inflammatory strategies. Additionally, targeting inflammation may offer adjunctive benefits in managing patients with diabetes, especially in those with obesity-related insulin resistance. Understanding species-specific immunometabolic responses will be essential for designing tailored therapies in both veterinary and human medicine.

## 3. Pharmacological Therapies

### 3.1. Insulin Therapies

#### 3.1.1. Brief Description

Insulin remains the cornerstone treatment for DM in both humans and veterinary medicine. However, significant differences exist in the types of insulin formulations used and the species-specific responses to these therapies.

#### 3.1.2. Key Findings

In humans, a wide array of insulin preparations is available, including rapid-acting (lispro and aspart), short-acting (regular insulin), intermediate-acting (neutral protamine Hagedorn [NPH]), and long-acting analogs (e.g., glargine, detemir) [69,70]. These options allow for individualized glycemic control strategies based on patient lifestyle, glucose patterns, and insulin sensitivity [71,72].

In veterinary medicine, insulin therapy is also the mainstay for managing diabetes in dogs and cats, but with more limited options. Dogs are commonly treated with Vetsulin (porcine lente insulin) or detemir [73,74]. Notably, canines show heightened sensitivity to detemir and often require significantly lower doses compared to humans [75]. Contrastingly, cats are most effectively managed using glargine or protamine zinc insulin (PZI) [76,77]. Glargine has been associated with diabetic remission in a substantial proportion of feline DM cases [76].

#### 3.1.3. Academic Discussion

Species-specific differences in insulin pharmacokinetics and pharmacodynamics play a critical role in determining the optimal insulin formulation, dosing, and administration frequency in diabetic management across humans, dogs, and cats.

For example, glargine insulin, a long-acting analog with a flat pharmacokinetic profile in humans, exhibits a shorter duration and less stable absorption in cats, requiring twice-daily injections to maintain glycemic control [78]. These differences are partly attributable to species-dependent variability in subcutaneous tissue perfusion, metabolic rate, and insulin receptor binding affinity, which alter absorption and clearance dynamics [26]. Similarly, detemir insulin demonstrates markedly higher potency in dogs due to stronger binding affinity to canine insulin receptors, necessitating significantly lower starting doses than those in humans [75]. This pharmacodynamic sensitivity underscores the importance of conservative dosing strategies and close blood glucose monitoring in canines [79]. The selection of insulin types in veterinary medicine is also influenced by formulation availability and ease of handling by pet owners. For instance, Vetsulin^®^ (porcine lente insulin) is commonly used in dogs due to its intermediate duration and ease of administration, whereas PZI is often preferred for cats because of its longer duration and compatibility with feline glucose dynamics [80].

Understanding these interspecies differences is essential for tailoring insulin regimens that balance efficacy, safety, and owner compliance. Furthermore, comparative research into insulin analog pharmacology can help bridge therapeutic gaps and guide the development of veterinary-specific formulations with improved pharmacokinetic profiles for long-term glycemic control.

### 3.2. Oral Hypoglycemic Agents

#### 3.2.1. Brief Description

Oral hypoglycemic agents are a mainstay for managing T2DM in humans, offering diverse mechanisms of action and flexible treatment options. However, their use in veterinary medicine is limited by species-specific differences in pathophysiology and drug tolerance.

#### 3.2.2. Key Findings

In humans, oral antidiabetic medications include biguanides (metformin), sulfonylureas, dipeptidyl peptidase-4 (DPP-4) inhibitors, GLP-1 receptor agonists, and SGLT2 inhibitors [81]. Each drug class targets different pathophysiological mechanisms, such as hepatic glucose production, pancreatic insulin secretion, incretin response, or renal glucose reabsorption [82]. Contrastingly, oral hypoglycemics are less effective or not routinely used in veterinary medicine. In cats, glipizide has been used off-label with moderate efficacy in early-stage diabetes; however, side effects such as GI upset and hepatotoxicity limit its long-term use [83]. Metformin is rarely recommended due to poor palatability, inconsistent absorption, and risk of lactic acidosis [84,85]. Dogs, which often present with insulin-dependent (type 1-like) diabetes, typically do not benefit from oral agents. Their β-cell function is usually significantly compromised at diagnosis, making insulin therapy essential [13].

#### 3.2.3. Academic Discussion

Although oral hypoglycemic agents revolutionized the treatment of T2DM in humans by targeting a broad spectrum of pathogenic pathways, their application in veterinary medicine remains highly constrained by species-specific differences in disease mechanisms, pharmacokinetics, and drug tolerability.

For instance, biguanides such as metformin are widely used in humans to suppress hepatic gluconeogenesis and improve insulin sensitivity. However, in cats and dogs, their utility is limited by the risk of lactic acidosis, GI side effects, and inconsistent bioavailability—exacerbated by species-specific digestive physiology [84,85]. Furthermore, glipizide, a sulfonylurea that stimulates pancreatic β-cell insulin secretion, shows limited efficacy in cats due to underlying β-cell amyloidosis and the progressive nature of feline diabetes [39]. Its use is also complicated by potential hepatotoxicity and GI intolerance [66].

In canine DM, which often resembles human T1DM, the severe β-cell destruction at diagnosis renders insulin secretagogues largely ineffective [35]. This difference in pathogenesis—insulin-dependent versus insulin-resistant states—highlights the reasons why insulin therapy remains essential in canine diabetes, and why oral agents are seldom effective [69].

Additionally, veterinary patients often exhibit variable drug absorption and metabolism, influenced by differences in gastric pH, enzymatic activity, and hepatic clearance, which may compromise the pharmacological profiles of oral agents developed for humans.

As novel classes of oral agents, such as SGLT2 inhibitors and GLP-1 receptor agonists, continue to expand therapeutic options in human medicine, translational studies are warranted to evaluate their safety, pharmacodynamics, and dosing regimens in companion animals. Tailored formulations and species-specific pharmacological trials may ultimately broaden the scope of oral hypoglycemic therapy in veterinary endocrinology.

### 3.3. Novel and Investigational Drugs

#### 3.3.1. Brief Description

Several novel and investigational therapies, particularly those targeting incretin pathways or novel mechanisms, are being explored across species.

#### 3.3.2. Key Findings

GLP-1 receptor agonists, such as liraglutide, demonstrated benefits in glycemic control and weight management in humans [86]. Recent pilot studies in cats suggest that these agents may improve glycemic regulation; however, long-term efficacy and safety remain under investigation [87,88].

SGLT2 inhibitors, which reduce renal glucose reabsorption, significantly improved glycemic management in humans [89]. Their potential in veterinary medicine is being explored, with preliminary studies in dogs demonstrating improved glycemic profiles and weight loss benefits [90,91]. However, species-specific renal physiology and risk of urinary tract infections must be carefully considered.

Amylin analogs such as pramlintide, used as adjuncts to insulin therapy in humans, remain largely unexplored in veterinary medicine [92,93]. Amylin plays a role in postprandial glucose regulation by slowing gastric emptying and suppressing glucagon secretion [94,95]. Despite its mechanistic promise, the lack of veterinary-specific formulations and limited clinical trials hinders its use in animals.

In addition to synthetic agents, natural plant-based extracts such as those from *Phlomis stewartii* have also shown promise in modulating oxidative and endoplasmic reticulum stress pathways in diabetic models, offering complementary avenues for adjunctive therapy [96].

#### 3.3.3. Academic Discussion

Although many of these therapeutic agents offer mechanistic advantages, their application in veterinary medicine remains limited. This is largely due to species-specific differences in drug metabolism, receptor profiles, and disease pathophysiology. For example, interspecies variation in GLP-1 receptor signaling and renal glucose transporter function may influence both drug efficacy and safety in dogs and cats compared to humans, although direct comparative data remain limited [62].

Additionally, clinical evidence in animals is often restricted to small-scale or experimental studies [62,97]. Regulatory and formulation challenges further constrain their veterinary use. Therefore, future research should prioritize pharmacokinetic and safety studies tailored to animal physiology and evaluate the feasibility of adapting successful human therapies for veterinary contexts.

Table 2 summarizes the availability, formulation challenges, and comparative application of major antidiabetic drugs across humans, dogs, and cats.

## 4. Pharmaceutical Considerations: Formulation and Drug Delivery

### 4.1. Species-Specific Challenges

#### 4.1.1. Brief Description

The development and administration of antidiabetic medications in humans, dogs, and cats face significant pharmaceutical challenges due to species-specific physiological and behavioral differences.

#### 4.1.2. Key Findings

Variability in GI physiology—gastric pH, transit time, enzymatic activity, and gut microbiota composition—affects oral drug solubility, stability, and bioavailability. Dogs exhibit faster gastric emptying than cats, influencing the performance of sustained-release formulations [98]. Cats have a shorter intestinal transit time and a higher intestinal pH, both of which may reduce the solubility and absorption of weakly basic drugs [99].

Sensory perception—odor, taste, and texture—also influences drug acceptance. Preservatives in insulin formulations, including phenol and m-cresol, may deter animals due to their heightened olfactory sensitivity. Particularly, cats often reject oral medications because of sensitivity to bitterness and texture, which reduces compliance and efficacy [100,101,102].

Behavioral stress from forced administration further exacerbates glycemic instability, especially in cats [103]. Therefore, palatable, low-stress formulations are essential for therapeutic success [104].

#### 4.1.3. Academic Discussion

Interspecies differences in drug absorption and response are rooted in complex physiological, metabolic, and behavioral traits that affect therapeutic outcomes. For instance, the higher intestinal pH in cats reduces drug solubility and alters the ionization states of certain compounds, thereby lowering membrane permeability and systemic bioavailability [99]. These pharmacokinetic limitations are compounded by behavioral challenges, such as stress-induced hyperglycemia or emesis, which further impair treatment efficacy. Moreover, the presence of specific drug transporters and metabolic enzymes may vary across species, influencing the absorption, distribution, and elimination of medications [105]. Understanding these multidimensional factors is vital for optimizing drug formulations, minimizing adverse effects, and improving long-term glycemic control in veterinary patients. Table 3 summarizes key pharmacokinetic parameters of common antidiabetic agents in humans, dogs, and cats.

### 4.2. Formulation Strategies

#### 4.2.1. Brief Description

Innovative pharmaceutical strategies are essential to overcome species-related limitations and enhance both therapeutic adherence and drug efficacy in diabetes management.

#### 4.2.2. Key Findings

Sustained-release insulin formulations, including depot injections or crystalline insulin zinc suspensions, demonstrated success in prolonging glycemic control in humans and are gradually being adapted for veterinary use. For example, PZI and porcine lente insulin formulations such as Vetsulin^®^ have been tailored to approximate the glucose profiles of canines and felines, although their duration of action remains highly variable among individual patients [80,106].

Implantable insulin pumps and closed-loop delivery systems (artificial pancreas) revolutionized human diabetes management. Although these delivery systems are not yet widely adopted in veterinary medicine, they offer promising translational applications, particularly in dogs with diabetes or among research or high-value animals, where real-time glucose monitoring and precision insulin delivery can be life-saving [107,108,109].

Nanotechnology-based drug delivery systems, such as lipid nanoparticles, polymeric micelles, and dendrimers, have the potential to improve the oral bioavailability of peptide-based drugs such as GLP-1 analogs. These technologies protect therapeutic peptides from enzymatic degradation in the GI tract. Microneedle patches also represent a promising transdermal delivery approach that may be applicable to both humans and animals, providing minimally invasive and sustained insulin administration [110,111].

Transmucosal delivery routes, including buccal and intranasal delivery, are actively being explored in veterinary medicine to bypass hepatic first-pass metabolism and potentially enhance systemic bioavailability. In cats, buccal administration of certain sedatives and analgesics has been successfully implemented, suggesting feasibility for future antidiabetic therapies [112].

#### 4.2.3. Academic Discussion

While traditionally underutilized in veterinary settings, novel delivery strategies hold significant potential to improve outcomes by addressing the pharmacokinetic and behavioral challenges inherent in animal patients. For example, nanocarriers not only stabilize peptides such as GLP-1, but also enable controlled release and targeted delivery, which may reduce dosing frequency and improve owner compliance [113]. Additionally, microneedle and transmucosal systems offer minimally invasive routes that bypass GI degradation and enhance patient comfort. Particularly, intranasal insulin demonstrated neuroprotective effects in human models of vascular dementia through transcriptomic and metabolomic modulation [114], indicating a broader therapeutic horizon. These advanced platforms highlight the need for species-specific innovation to translate cutting-edge pharmaceutical technology into practical veterinary applications.

### 4.3. Compliance and Owner Considerations

#### 4.3.1. Brief Description

Owner-related factors and animal compliance are critical determinants of therapeutic success in veterinary diabetic care. Unlike human patients, animals rely entirely on caregivers for consistent medication administration, and their behavioral responses to therapy can significantly influence outcomes [115].

#### 4.3.2. Key Findings

Noncompliance is frequently reported due to difficulties in handling, refusal to ingest oral medications, and stress associated with repeated injections. These challenges are particularly pronounced in cats, where daily insulin injections and poor palatability of oral formulations lead to treatment discontinuation or euthanasia decisions in severe cases [115,116].

Formulation innovations can substantially improve adherence. Palatable, chewable tablets, flavored suspensions, and once-daily long-acting insulin analogs are examples of design features that enhance owner convenience and animal cooperation [79,104]. Moreover, drug delivery systems with reduced frequency of administration (weekly or monthly depot injections) are highly desirable in veterinary settings where repeated veterinary visits are impractical or stressful for the animal [117].

#### 4.3.3. Academic Discussion

Improving therapeutic compliance requires a multifaceted approach that addresses both animal behavior and caregiver burden. Educating pet owners about the importance of adherence, while providing user-friendly drug delivery systems, can significantly enhance treatment outcomes [102,118]. Furthermore, pharmaceutical development in veterinary endocrinology must consider pharmacological efficacy and behavioral science and human–animal interaction dynamics [119]. By integrating clinical pharmacology with practical home-administration strategies, future therapies can better meet the needs of both patients and caregivers.

## 5. Regulatory and Clinical Approval Status

The regulatory landscape for antidiabetic therapies varies significantly between human and veterinary medicine, reflecting differences in disease prevalence, species-specific pharmacodynamics, and market demands. Understanding these distinctions is critical for translational research, clinical application, and ethical prescribing in veterinary endocrinology.

### 5.1. Human-Approved Antidiabetic Agents

#### 5.1.1. Key Findings

In human medicine, antidiabetic therapies undergo rigorous evaluation by regulatory agencies such as the U.S. Food and Drug Administration (FDA) and the European Medicines Agency (EMA). These agencies approved a wide range of therapeutic agents to manage DM, encompassing multiple pharmacological classes.

In addition to insulin, several classes of oral hypoglycemic agents are approved, including metformin (a biguanide), sulfonylureas, meglitinides, and thiazolidinediones [120]. Furthermore, incretin-based therapies emerged as valuable tools, including DPP-4 inhibitors such as sitagliptin and linagliptin, and GLP-1 receptor agonists such as liraglutide and semaglutide [121].

SGLT2 inhibitors, such as empagliflozin and dapagliflozin, also gained widespread approval for their dual effects on glycemic control and cardiovascular risk reduction [122].

These medications undergo comprehensive regulatory review, which includes evaluations of their efficacy, pharmacokinetic properties, manufacturing consistency, and long-term safety profiles, particularly with respect to cardiovascular outcomes, renal effects, and carcinogenic potential [123,124].

#### 5.1.2. Academic Discussion

Treatment strategies and medication approvals for DM differ across humans, dogs, and cats due to species-specific physiological, genetic, and pharmacokinetic differences. Dogs typically develop insulin-dependent DM resembling T1DM, often associated with pancreatic β-cell destruction [35]. Contrastingly, cats more frequently exhibit a type 2-like phenotype, characterized by insulin resistance and islet amyloidosis [39,125]. These underlying differences necessitate tailored treatment approaches, including species-specific insulin formulations and dosing regimens.

Furthermore, interspecies variability in hepatic metabolism, insulin receptor sensitivity, and cytochrome P450 activity can influence pharmacokinetics and pharmacodynamics. For instance, cats generally exhibit slower drug clearance and prolonged insulin action, favoring the use of longer-acting formulations such as PZI [77]. Contrastingly, dogs may require more frequent administration due to the faster metabolism of some agents [126].

### 5.2. Veterinary-Approved Antidiabetic Agents

#### 5.2.1. Key Findings

The repertoire of FDA- or EMA-approved antidiabetic drugs in veterinary medicine remains limited, primarily due to species-specific pharmacological responses, smaller commercial markets, and ethical constraints in conducting large-scale clinical trials in animals.

Currently, only a few insulin products received regulatory approval specifically for use in veterinary patients. Vetsulin^®^ (porcine insulin zinc suspension, 40 IU/mL) is approved by the United States FDA under NADA 141-236 for the treatment of DM in both dogs and cats [127]. In the European Union, the same formulation is marketed as Caninsulin^®^ and received marketing authorization from the EMA for use in dogs and cats [128].

Another product, ProZinc, a PZI formulation, was initially approved by the FDA (NADA 141-294) for use in cats with diabetes and was subsequently approved for use in dogs by the FDA in 2019 [80,129,130].

These veterinary insulin formulations are developed with species-specific pharmacokinetic characteristics in mind and include dosing recommendations tailored to dogs and cats. Nevertheless, the pharmacodynamic responses to these insulins can vary widely between individual patients, requiring careful dose titration and regular blood glucose monitoring to achieve optimal glycemic control [106,131].

As of 2022, no oral hypoglycemic agents had been formally approved by the FDA or EMA for the treatment of diabetes in dogs or cats, with the exception of bexagliflozin (Bexacat), which was recently authorized by the FDA exclusively for feline use [132]. Consequently, the management of feline type 2-like diabetes and insulin-resistant diabetes in dogs continues to rely almost exclusively on insulin-based therapy [133,134].

#### 5.2.2. Academic Discussion

The reliance on insulin-based therapy in dogs and cats with diabetes reflects biological factors, such as species-specific pharmacokinetics, and regulatory limitations, as few antidiabetic agents received formal approval for veterinary use [133,134]. Unlike human medicine, where robust clinical trials and extensive funding support the development of diverse pharmacologic classes, veterinary drug development is constrained by limited scale, funding, and ethical feasibility [135,136]. Moreover, the metabolic and physiological differences between dogs, cats, and humans require dedicated pharmacokinetic and pharmacodynamic studies to ensure the safe and effective use of antidiabetic agents [137].

Additionally, the heterogeneity in insulin responsiveness among individual animals adds complexity to therapeutic standardization. For instance, while ProZinc and Vetsulin offer tailored options, their onset and duration of action can be unpredictable, necessitating personalized titration and close monitoring of glycemic trends [106,138]. This highlights the importance of real-world evidence collection and post-approval surveillance in veterinary endocrinology.

To expand treatment options, regulatory frameworks must adapt to encourage innovation while ensuring animal safety [139]. This could involve incentives for pharmaceutical companies to invest in veterinary drug trials, the validation of alternative study designs, and cross-species translational research. Importantly, enhanced collaboration between academia, industry, and regulatory authorities will be vital to overcoming current limitations and advancing diabetes care in veterinary medicine.

### 5.3. Off-Label Use and Regulatory Considerations

#### 5.3.1. Key Findings

Given the limited availability of species-specific pharmaceuticals, the off-label use of human-approved medications in veterinary patients is relatively common. However, such practices carry important regulatory and ethical considerations. In the United States, the Animal Medicinal Drug Use Clarification Act provides a legal framework under which veterinarians may prescribe drugs in an extra-label manner. Similar regulatory provisions exist in the European Union.

Under these guidelines, off-label prescribing is permissible only when specific conditions are met. These include the existence of a valid veterinarian–client–patient relationship, the unavailability of an approved veterinary alternative, and assurance that the drug’s use does not pose unacceptable risks to the animal or to public health [140].

This approach is particularly relevant to oral antidiabetic agents such as glipizide (a sulfonylurea) and metformin, both of which have been experimentally investigated in feline diabetes, with limited and variable success. However, these medications may induce adverse effects, including GI disturbances and hypoglycemia, and often exhibit inconsistent or unpredictable efficacy in cats. Therefore, their use requires caution and close monitoring [84,141].

#### 5.3.2. Academic Discussion

Off-label drug use in veterinary endocrinology underscores a critical intersection between necessity and responsibility. While such prescribing practices may expand therapeutic options for animals with diabetes, they must be grounded in sound clinical rationale, species-specific knowledge, and appropriate ethical safeguards. A clear understanding of pharmacokinetic profiles, metabolism, and species-dependent receptor interactions is essential before extrapolating human drug data to veterinary patients [26,142].

For instance, glipizide and metformin—though commonly used in human T2DM—show inconsistent absorption and efficacy in cats, potentially leading to under- or overtreatment [84,141]. These concerns are compounded by the absence of formal dosing guidelines and long-term safety data for such agents in veterinary populations [134,143]. Consequently, veterinarians must exercise judicious decision-making, rely on published evidence where available, and ensure that informed consent is obtained from pet owners.

Inappropriate off-label use not only risks poor clinical outcomes and animal distress, but may also result in reputational and legal consequences for practitioners [144]. To address these challenges, enhanced post-market surveillance, continuing education on off-label protocols, and the development of centralized databases documenting extra-label use outcomes would be beneficial. Ultimately, fostering a culture of evidence-based and ethically justified off-label prescribing will improve diabetes care in veterinary medicine.

### 5.4. Emerging Approvals and Translational Perspectives

#### 5.4.1. Key Findings

Recent advancements in diabetes therapeutics introduced several novel agents and delivery platforms in human medicine, including long-acting insulin analogs, GLP-1 receptor agonists, and SGLT2 inhibitors. Although these innovations are not yet formally approved for use in veterinary species, they hold considerable promise for translational application in dogs and cats [132,145].

A notable example is the development of ultra-long-acting insulin formulations that enable once-weekly dosing. Among these, AKS-218d, a recombinant fusion insulin analog, has been evaluated in dogs with diabetes and demonstrated sustained glycemic control with a single subcutaneous injection per week. Preliminary data from a recent study demonstrated that dogs treated with AKS-218d maintained stable fructosamine levels and showed clinical improvement similar to that observed with conventional twice-daily insulin regimens, with no significant adverse effects reported [117].

Such depot-based insulin formulations are particularly advantageous in veterinary practice, where frequent injections can be stressful for animals and challenging for caregivers. Reducing the frequency of administration has the potential to improve compliance, minimize clinic visits, and enhance the overall quality of care in pets with diabetes [146].

#### 5.4.2. Academic Discussion

While translational innovation from human to veterinary medicine offers great promise, several scientific and regulatory hurdles must be addressed before these therapies can be widely implemented in companion animals. First, species-specific differences in drug absorption, distribution, metabolism, and excretion must be characterized to define safe and effective dosing strategies [26,142]. Second, large-scale clinical trials in veterinary populations are required to establish evidence of efficacy and safety, particularly across diverse breeds, age groups, and comorbid conditions [147].

Regulatory approval will also hinge on the availability of species-specific pharmacokinetic and pharmacodynamic data [148]. Current gaps in long-term outcome studies and adverse event monitoring hinder the progression of investigational agents into routine clinical use [149]. Moreover, the high cost of novel therapies may limit accessibility for pet owners, further underscoring the need for cost–benefit analyses tailored to the veterinary context [150,151].

To facilitate translation, enhanced collaboration among academic institutions, industry sponsors, and regulatory agencies is critical. Strategic initiatives, such as adaptive trial designs, shared data repositories, and harmonized approval pathways, can expedite the integration of innovative therapies into veterinary practice. Ultimately, bridging the gap between experimental promise and clinical reality will require a robust infrastructure that supports responsible, evidence-based innovation in veterinary endocrinology.

## 6. Future Directions and Translational Implications

Comparative studies on DM in humans, dogs, and cats highlight shared and divergent pathophysiological features, offering valuable opportunities for translational research that can benefit both veterinary and human medicine [1].

### 6.1. Cross-Species Insights for Translational Research

Canine and feline diabetes models reflect key features of human diabetes, particularly in relation to insulin resistance, β-cell dysfunction, and glucose homeostasis dysregulation. For example, feline diabetes closely resembles human T2DM, with obesity-related insulin resistance and islet amyloid deposition [152]. Conversely, canine diabetes often mimics T1DM, with progressive β-cell destruction and insulin dependency [35].

This species-specific divergence offers a unique platform for exploring mechanisms of disease progression, response to insulin analogs, and early detection of therapeutic failure. Notably, GLP-1 analogs, SGLT2 inhibitors, and DPP-4 inhibitors have been explored in feline and canine models, potentially providing translational insights applicable to human diabetes therapy [61,132].

#### Academic Discussion

By leveraging spontaneous disease models in companion animals, researchers can investigate pathophysiological mechanisms under naturally occurring conditions that better reflect the clinical heterogeneity of human diabetes [11]. This approach offers insight into how different pathological mechanisms, such as inflammation, islet amyloidosis, or glucotoxicity, manifest across species [11]. Furthermore, studying comparative responses to therapeutic agents helps identify biomarkers predictive of treatment efficacy or failure [153].

### 6.2. Veterinary Research Informing Human Therapies

Veterinary patients naturally develop diabetes and offer long-term disease models without the need for artificial induction, thereby reducing ethical and methodological limitations associated with rodent models [10]. For instance, long-acting insulins, such as glargine, have been used successfully in cats with diabetes, contributing to remission in some cases. Remission observed in feline diabetes following glargine treatment sparked interest in the mechanisms of β-cell preservation, which may inform similar strategies in human medicine [76].

Furthermore, the use of continuous glucose monitoring in dogs and cats with diabetes provides real-world data on glucose fluctuation patterns, aiding the refinement of insulin dosing algorithms applicable to pediatric and older adult populations with diabetes in human medicine [14,154].

#### Academic Discussion

Companion animal models generate clinically relevant data under real-world conditions, supporting the external validity of experimental findings [155]. Veterinary insights into dose titration, glucose variability, and patient adherence offer opportunities to refine human diabetes care, particularly for populations with similar challenges, such as children or cognitively impaired adults [156]. Moreover, studying disease remission and relapse in animals contributes to the understanding of β-cell resilience and endocrine plasticity [38].

### 6.3. Companion Animals as Preclinical Models

Companion animals with spontaneous diabetes serve as clinically valuable preclinical models for evaluating drug efficacy, pharmacokinetics, and delivery systems. Unlike rodent models, companion animals share similar living environments, dietary habits, and circadian rhythms with humans, making them ideal models for evaluating long-term therapeutic efficacy and adherence behaviors [157].

Moreover, advances in noninvasive delivery platforms (e.g., transdermal insulin, palatable GLP-1 formulations) in veterinary settings can inform human pharmaceutical innovation, particularly for pediatric and geriatric patients with injection aversion or dysphagia [111,158].

#### Academic Discussion

In addition to replicating clinical heterogeneity, companion animals facilitate longitudinal studies on chronic medication exposure, adherence, and comorbidities [159]. These models help evaluate the practicality and durability of new therapies in real-world settings [159]. Veterinary adaptation of novel drug delivery technologies, such as microneedles or transmucosal systems, also accelerates human drug development by addressing shared challenges in drug administration and patient compliance [111].

### 6.4. Integrated One Health Approach

**Figure 2:**
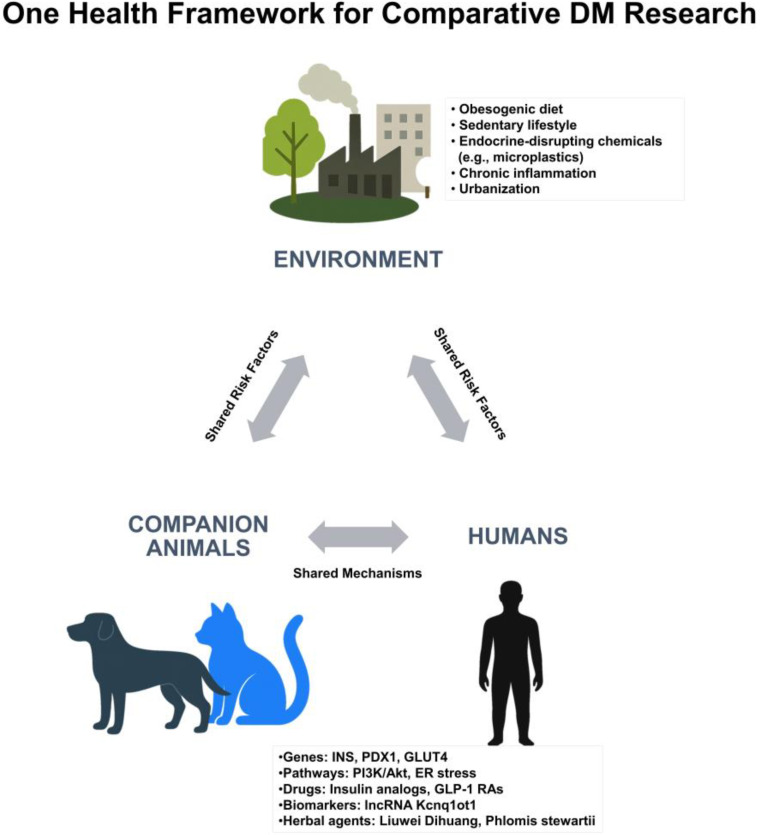
One Health framework illustrating shared mechanisms and risk factors among humans, companion animals, and the environment in the context of DM. ER, endoplasmic reticulum; PI3K/Akt: phosphoinositide 3-kinase/protein kinase B signaling pathway; and lncRNA, long noncoding RNA.

Adopting a One Health approach that bridges veterinary and human medicine encourages collaborative research in endocrinology, pharmacology, and drug development. A conceptual summary of the One Health framework, highlighting shared mechanisms and environmental risk factors in comparative diabetes research, is presented in Figure 2.

Recent findings identified long non-coding RNAs, such as *Kcnq1ot1*, as emerging biomarkers in T2DM and its comorbidities, offering novel diagnostic and therapeutic perspectives that may be relevant across species [160]. Shared metabolic and hormonal mechanisms across species provide a solid foundation for the co-development of therapeutic strategies and the discovery of biomarkers for early diagnosis, treatment response, and monitoring [161].

Recent comparative genomic analyses revealed conserved and species-specific variants in key diabetes-associated genes, such as *INS*, *PDX1*, and *GLUT4*, across humans, dogs, and cats. Incorporating these data may provide further insight into the molecular pathogenesis of DM across species. A comparative summary of gene conservation and function is presented in Table 4.

## 7. Conclusions

DM presents with both overlapping and species-specific characteristics across humans, dogs, and cats. These interspecies differences, ranging from autoimmune β-cell destruction in dogs to insulin resistance and islet amyloidosis in cats, highlight the need for tailored therapeutic strategies that reflect each species’ unique pathophysiology [157,162].

While human medicine offers a broad spectrum of well-characterized antidiabetic therapies supported by extensive clinical trials and regulatory systems, veterinary medicine remains limited by a narrow formulary, off-label reliance on human drugs and formulation challenges [16,163]. Species-specific variations in pharmacokinetics, metabolism, and behavior, such as stress-induced hyperglycemia in cats or poor compliance with oral medications, necessitate not only dose adjustments, but also a re-evaluation of delivery systems [164,165].

Recent advances in veterinary pharmacotherapy, such as long-acting insulin analogs and minimally invasive delivery platforms (e.g., microneedle patches, buccal sprays), demonstrate that innovation in animal care can address unmet clinical needs while simultaneously offering translational insights [166]. These technologies are particularly relevant to vulnerable human populations, including pediatric, geriatric, or needle-averse patients, for whom conventional delivery systems may be inadequate.

Recognizing companion animals as spontaneous, naturally occurring models of diabetes provides a valuable bridge between experimental findings and real-world clinical application [10]. Unlike induced rodent models, dogs and cats with diabetes offer greater translational fidelity in disease progression, therapeutic response, and environmental exposures.

To fully leverage this potential, future efforts must embrace the One Health framework, where discoveries in veterinary patients not only improve animal care, but also inform and accelerate innovation in human diabetes treatment. Cross-disciplinary collaboration among veterinarians, physicians, pharmaceutical scientists, and regulators will be essential to building a bi-directional flow of knowledge. Such integrative approaches will enable the development of species-appropriate therapies while driving therapeutic breakthroughs that benefit both human and veterinary medicine.

## Figures and Tables

**Table 1:** Comparative pathophysiology of diabetes mellitus across humans, dogs, and cats.

Species	Dominant Diabetes Type	Pathophysiological Features	Hormonal Responses	Key Notes	Symptoms
Humans	Type 1 and type 2	Autoimmune β-cell loss (T1); insulin resistance, β-cell dysfunction, amyloid (T2)	GLP-1, amylin, insulin, glucagon	Type 2 is prevalent in adults; multifactorial origins	Polyuria, polydipsia, weight loss, fatigue, blurred vision
Dogs	Type 1-like	β-cell destruction (nonautoimmune); insulin-dependent	Limited studies; possible GLP-1 analog effects	Middle-aged to older onset; irreversible	Polyuria, polydipsia, weight loss, increased appetite, cataracts
Cats	Type 2-like	Insulin resistance, islet amyloidosis, and obesity-related inflammation	Amylin overexpression, glucagon dysregulation	Remission possible; strong dietary influence	Polyuria, polydipsia, weight loss, hindlimb weakness, lethargy

Abbreviations: GLP-1, glucagon-like peptide-1; T1, type 1; and T2, type 2.

**Table 2:** Species-specific pharmacological and pharmaceutical considerations for antidiabetic therapy.

Aspect	Humans	Dogs	Cats	Comparative Notes	Representative Drugs (Chemical Name/Formula)
Preferred insulin	Lispro, Glargine, Detemir, Degludec	Vetsulin, Detemir	Glargine, PZI	Glargine is effective across species; detemir dose differs	Insulin glargine (C267H404N72O78S6), insulin lispro (C257H383N65O77S6)
Oral agents	Metformin, SGLT2i, DPP-4i, GLP-1 RA	Rare; limited metformin use	Rare; some metformin off-label	Limited bioavailability and safety data in animals	Metformin HCl (C4H11N5·HCl), empagliflozin (C23H27ClO7), Liraglutide (MW, approximately 3751 Da)
Formulation challenges	Tablet, injection, pens	Injection (owners trained)	Injection; low oral compliance	Cats poorly tolerate oral meds; stress worsens glycemia	Oral tablets (metformin), solution pens (liraglutide), long-acting injections
PK variability	Well-characterized	Variable absorption	Slow clearance of some agents	Enzymatic, GI differences affect dosing	Bioavailability: metformin, approximately 50–60%; GLP-1 RAs, <1% oral

Abbreviations: SGLT2i, sodium-glucose co-transporter 2 inhibitor; DPP-4i, dipeptidyl peptidase-4 inhibitor; GLP-1 RA, glucagon-like peptide-1 receptor agonist; PZI, protamine zinc insulin; GI, gastrointestinal; and PK, pharmacokinetics.

**Table 3:** Comparative pharmacokinetic characteristics of antidiabetic drugs in humans, dogs, and cats.

Drug/Class	Species	Route	Tmax (h)	T½ (h)	Bioavailability (%)	Notes
Insulin glargine	Human	SC	1–2	12–24	approximately 60–80	Peakless profile
Insulin glargine	Cat	SC	2–4	~12	Variable	May require BID dosing
Metformin	Human	Oral	2–3	4–8	50–60	Food delays Tmax
Metformin	Cat	Oral	3–4	4–6	Low	Poor palatability

Abbreviations: SC, subcutaneous; Tmax, time to maximum plasma concentration; T½, elimination half-life; and BID, twice daily.

**Table 4:** Comparative conservation of diabetes-associated genes in humans, dogs, and cats.

Gene	Function	Human	Dog	Cat
*INS*	Insulin synthesis and secretion	Highly conserved	Conserved	Conserved
*PDX1*	Pancreatic β-cell development and insulin gene transcription	Highly conserved	Conserved	Conserved
*GLUT4* (*SLC2A4*)	Facilitation of glucose transport in muscle and adipose tissue	Highly conserved	Moderately conserved	Moderately conserved
*KCNJ11*	ATP-sensitive potassium channel regulation of insulin release	Common SNPs linked to T2DM	Limited polymorphism data	Limited data
*HNF1A*	Transcription factor involved in β-cell function	Mutations linked to MODY3	Functional orthologs present	Functional orthologs present

Abbreviations: SNP, single nucleotide polymorphism; T2DM, Type 2 diabetes mellitus; and MODY3, maturity-onset diabetes of the young type 3.

## Data Availability

No new data were created or analyzed in this study. Data sharing is not applicable to this article.

## Abbreviations

The following abbreviations are used in this manuscript:

CGM	Continuous glucose monitoring
DM	Diabetes mellitus
DPP-4	Dipeptidyl peptidase-4
EMA	European Medicines Agency
FDA	Food and Drug Administration
GI	Gastrointestinal
GLP-1	Glucagon-like peptide-1
GLP-1 RA	Glucagon-like peptide-1 receptor agonist
IAPP	Islet amyloid polypeptide
MODY3	Maturity-onset diabetes of the young type 3
NPH	Neutral protamine hagedorn
PZI	Protamine zinc insulin
SGLT2	Sodium-glucose co-transporter 2
SNP	Single nucleotide polymorphism
T1DM	Type 1 diabetes mellitus
T2DM	Type 2 diabetes mellitus
